# A 4-Week Repeated-Dose Oral Toxicity Study of Bojungikgi-Tang in Crl:CD Sprague Dawley Rats

**DOI:** 10.1155/2017/4748904

**Published:** 2017-12-13

**Authors:** Sae-Rom Yoo, Hyekyung Ha, Mee-Young Lee, Hyeun-Kyoo Shin, Su-Cheol Han, Chang-Seob Seo

**Affiliations:** ^1^K-Herb Research Center, Korea Institute of Oriental Medicine, 1672 Yuseong-daero, Yuseong-gu, Daejeon 34054, Republic of Korea; ^2^Jeonbuk Department of Inhalation Research, Korea Institute of Toxicology, 30 Baehak 1-gil, Jeongeup-si, Jeollabuk-do 56212, Republic of Korea

## Abstract

Traditional herbal medicines have been used for centuries in Asian countries. However, recent studies have led to increasing concerns about the safety and toxicity of herbal prescriptions. Bojungikgi-tang (BJIGT), a herbal decoction, has been used in Korea to improve physical strength. To establish the safety information, BJIGT water extract was evaluated in a 4-week repeated-dose oral toxicity test in Crl:CD Sprague Dawley rats. BJIGT was orally administered in daily doses of 0, 500, 1000, and 2000 mg/kg/day for 4 weeks via oral gavage in male and female rats. We examined the mortality, clinical signs, body weight change, food intake, organ weights, hematology, serum biochemistry, and urinalysis parameters. No significant changes were observed in mortality, clinical sings, body weight, food intake, organ weights, hematology, serum biochemistry, and urinalysis parameters between the control group and the BJIGT-treated groups in the rats of both sexes. The results indicate that BJIGT did not induce toxic effects at a dose level up to 2000 mg/kg in rats. Thus, this concentration is considered the nonobservable effect dose in rats and is appropriate for a 13-week subchronic toxicity study.

## 1. Introduction

Herbal medicine has increased in popularity over the last two decades. As the use of herbal medicine has increased gradually, concerns are raised about the safety and toxicity of herbal prescriptions [1, 2]. Although herbal medicine has been proven to be safe through clinical experience obtained from clinical herbalist, safety continues to be a major issue in the use of herbal medicines.

A traditional herbal medicine, Bojungikgi-tang (BJIGT, Bu Zhong Yi Qi Tang in Chinese and Hochuekkito in Japanese), has been used for centuries in Korea to improve digestive disorder including gastroptosis, myasthenia gastrica, and leiasthenia [3]. BJIGT is ranked fifth out of 56 herbal formulas covered by national health insurance in terms of medical expenses, which means that it is the most commonly used herbal formula in traditional Korean medicine [4, 5]. Recent pharmacological studies reported that BJIGT restored metabolic changes against virus infection [6, 7] and enhances cisplatin-induced apoptosis [8]. However, the safety of BJIGT is not yet fully elucidated.

Here we investigated the 4-week repeated-dose oral toxicity of BJIGT in Crl:CD Sprague Dawley (SD) rats. Through this study, we aimed to evaluate its safety and toxicity including information about the no-observed-adverse-effect level (NOAEL).

## 2. Materials and Methods

### 2.1. Preparations of BJIGT Decoction

Bojungikgi-tang water extract was manufactured by Sungil Bioex Co., Ltd. (Hwaseong, Korea). Briefly, eight component herbal medicines of BJIGT, Astragali Radix (36.885 kg), Glycyrrhizae Radix et Rhizoma (24.590 kg), Ginseng Radix (24.590 kg), Atractylodis Rhizoma Alba (24.590 kg), Citri Unshius Pericarpium (12.295 kg), Angelicae Gigantis Radix (12.295 kg), Cimicifugae Rhizoma (7.377 kg), and Bupleuri Radix (7.377 kg), were purchased from Kwangmyungdang Medicinal Herbs (Ulsan, Korea), mixed, and extracted in a 10-fold mass (1,500 L) of water at 100°C for 2 h using the reflux method. The extracted water solution was freeze-dried to give a powder (extract amount: 47.0 kg, yield: 31.3%).

### 2.2. High-Performance Liquid Chromatography Analysis of BJIGT Samples

Liquiritin (99.6%) and glycyrrhizin (99.1%) were purchased from Wako Chemicals (Osaka, Japan). Hesperidin (98.6%) and nodakenin (98.0%) were purchased from Biopurify Phytochemicals (Chengdu, China) and NPC BioTechnology (Yeongi, Korea). The standard solution of each reference standard was prepared at a concentration of 1000.0 *μ*g/mL using methanol and stored in the refrigerator. The conditions for HPLC analysis of BJIGT samples were described in detail in previous research [9]. Briefly, the high-performance liquid chromatography (HPLC) system used in this analysis was a Shimadzu Prominence LC-20A series (Kyoto, Japan) coupled with a photodiode array detector. The column used was SunFire C18 (250 mm × 4.6 mm; 5 *μ*m, Waters, Milford, MA, USA), and it was maintained at 40°C. The mobile phase system consisted of distilled water and acetonitrile, both with 1.0% (v/v) acetic acid.

### 2.3. Animals and Maintenance

This study was performed in the Korea Institute of Toxicology (earned AAALAC International accreditation in 1998). The study protocol was approved by the Institutional Animal Care and Use Committee according to the “Guidelines for Toxicity Tests for Drugs and Related Materials, Document #2015-82” as prepared by the Korean Ministry of Food and Drug Safety.

5-week-old Crl:CD SD rats of each sex were obtained from Orient Bio Co. (Seongnam, Republic of Korea). Three animals were allocated per stainless-steel cage for the observation period. All animals were housed under controlled conditions of temperature (23°C ± 3°C) and relative humidity (50%  ± 10%) with a 12 h light/dark cycle, a light intensity of 150–300 Lux, and 10–20 air changes per hour. They were fed a rodent diet (catalog number: 5053, PMI Nutrition International LLC., MO, USA) and sterilized tap water ad libitum.

### 2.4. Group Assignment and Experimental Treatment

Based on the prior single-oral-dose study of BJIGT [5], the highest dose level of 2000 mg/kg was chosen for the subacute toxicity. Using the Pristima system (Version 6.4.1, Xybion Medical System Co., NJ, USA), healthy animals were randomly assigned to four groups (*n* = 5/group): BJIGT 500, 1000, and 2000 mg/kg/day groups and a vehicle control group. BJIGT was prepared prior to each administration, and the daily administration volume (10 mg/kg body weight) was calculated based on the recorded recent body weight of each animal. The vehicle control group received an equal volume of distilled water.

Clinical signs and mortality were monitored twice a day. Body weight and food consumption were recorded twice a week. Daily food intake was measured per cage and calculated as average daily intake per mouse.

### 2.5. Necropsy

All the animals were anesthetized by isoflurane and then euthanized by exsanguination from the postcaval vein and aorta. Absolute organ weights were measured and relative organ weights were calculated as a ratio for the following organs: brain, heart, lung, kidneys, liver, spleen, reproductive organs, thymus, thyroid and parathyroid glands, pituitary gland, adrenal gland, seminal vesicles with coagulating gland, and salivary glands.

### 2.6. Hematology, Serum Biochemistry, and Urinalysis

Animals were fasted over a 16 h period prior to blood collection. Blood samples were collected in a collection tube containing 3.2% sodium citrate and were analyzed to determine white blood cell (WBC), total red blood cell (RBC), hemoglobin (HGB), hematocrit (HCT), mean corpuscular volume (MCV), mean corpuscular hemoglobin (MCH), mean corpuscular hemoglobin concentration (MCHC), platelet count (PLT), prothrombin time (PT), activated partial thromboplastin time (APTT), and large unstained cells (LUC) using the ADVIA 2120i hematology analyzer (Siemens Healthcare, Erlangen, Germany) and the ACL Elite Pro coagulation analyzer (Instrumentation Laboratory, Milan, Italy).

Serum samples were used to analyze clinical chemistry parameters including blood urea nitrogen (BUN), creatine kinase (CK), albumin/globulin ratio (AG), aspartate aminotransferase (AST), alanine aminotransferase (ALT), gamma glutamyl transpeptidase (GGT), total bilirubin (TB), total cholesterol (TCHO), triglyceride (TG), phospholipid (PL), and alkaline phosphatase (ALP) with a Toshiba 120 FR chemistry analyzer (Toshiba Co., Tokyo, Japan).

During the last week of treatment, urine samples were collected at approximately 16 h in metabolic cages, and urinalysis was conducted using the Cobas U 411 urine analyzer (Roche, Berlin, Germany) and Combur 10 TM urine sticks (Roche). Analysis included urine volume, specific gravity, and pH.

### 2.7. Statistical Analysis

Data are expressed as mean ± standard deviation. Bartlett's test was used to test the homogeneity of variances. When data have equal variance, group differences were assessed by a one-way analysis of variance (ANOVA) and a post hoc Dunnett's test using Prisma System. When data variances are not equal, group differences were assessed by the Kruskal-Wallis test and a post hoc Dunn rank-sum test.

## 3. Results 

### 3.1. HPLC Analysis of BJIGT Sample

**Figure 1:**
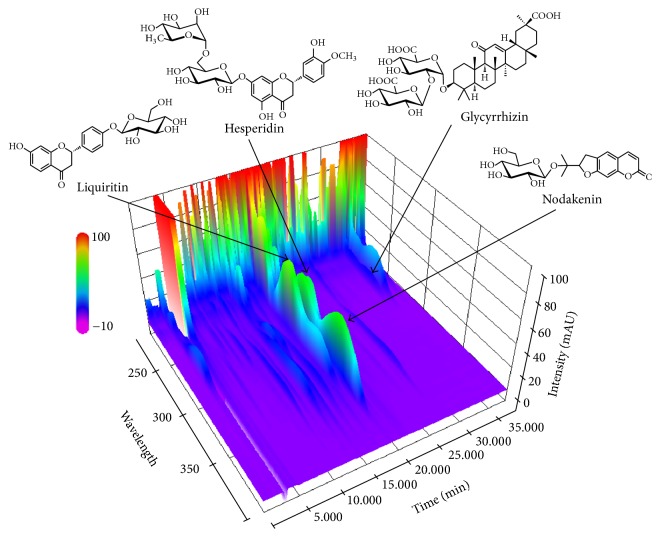
Three-dimensional HPLC chromatogram of the BJIGT samples using HPLC-PDA.

Simultaneous analysis of four major marker components in the BJIGT sample was performed using the optimized HPLC analytical method. As a result, four marker compounds, liquiritin, nodakenin, hesperidin, and glycyrrhizin, were detected at 17.708, 18.604, 19.654, and 34.259 min, respectively (Figure 1). At 0, 1, and 4 weeks in the BJIGT sample, the amount of liquiritin was 1.90 mg/g, that of nodakenin was 1.23–1.28 mg/g, that of hesperidin was 1.63–1.73 mg/g, and that of glycyrrhizin was 4.52–4.69 mg/g (Table 1).

### 3.2. Mortality and Clinical Signs

No deaths occurred in any of the animals during the 4 weeks of the experimental period (Table 2). A scratch wound was observed in two male rats in the vehicle control group (Table 2).

### 3.3. Changes in Body Weight and Food Intake

**Figure 2:**
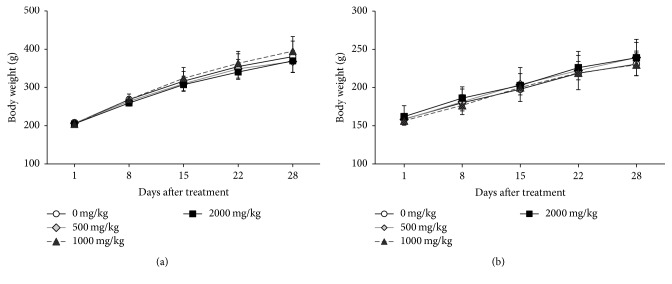
Body weights of male (a) and female (b) Crl:CD SD rats given the BJIGT. Results are presented as mean ± SD.

**Figure 3:**
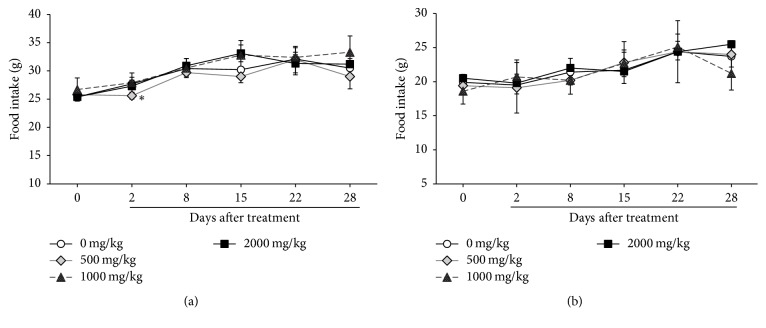
Food intakes of male (a) and female (b) Crl:CD SD rats given the BJIGT. Results are presented as mean ± SD. *∗* indicates significance at the *p* < 0.05 level.

The body weight of each rat increased in a time-dependent manner (Figure 2). However, BJIGT did not affect body weight changes in either sex of rats compared to the vehicle control group. Although food intake was decreased in male rats treated with BJIGT at 500 mg/kg/day following 2 days of treatment, the rats recovered after 6 days, and no significant differences were found during the experimental periods (Figure 3).

### 3.4. Necropsy Findings and Relative Organ Weights

Liver discoloration (*n* = 1; brown color in caudate lobe) was found in a male rat treated with BJITGT at 1000 mg/kg/day. Flaccid testes (*n* = 1, slight) were found in a male rat treated with BJIGT at 2000 mg/kg/day. However, these changes did not have a correlation with the dose of BJIGT. No significant changes were observed in relative organ weights (Table 6).

### 3.5. Hematology, Serum Biochemistry, and Urinalysis

There were no significant changes in hematology in either sex of rats treated with BJIGT compared with the vehicle control group (Table 3). In the female group, the levels of inorganic phosphorus (IP) and chloride (Cl) were increased at 500 mg/kg/day in the BJIGT-treated group (Table 4). The level of sodium (Na) was increased at 2000 mg/kg/day in the BJIGT-treated group. However, no effect of BJIGT was observed on serum biochemistry in the male group. In urinalysis, there was no significant difference between rats treated with BJIGT and the vehicle control group (Table 5).

## 4. Discussion

Herbal medicine is gaining popularity and is being marketed as alternative drugs or dietary supplements. Although herbal formulas are taken for a few days or longer periods of time based on an individual's status, many of them are not pharmaceutically tested to establish a toxic dose. Toxicity and side effects are closely related to drug dosage and duration of treatment. Previous study reported a safety evaluation of BJIGT using animal models. BJIGT had no toxic effects at doses up to 5 g/kg given within 24 hours in mice of both sexes [10].

Good Laboratory Practice (GLP) refers to the quality control process for nonclinical safety test [11]. Unlike preliminary study, repeated-dose toxicity study needs to comply with GLP regulation. This study was performed according to GLP standards to confirm the safety of BJIGT in Crl:CD SD rats. Consistent with previous results, there were no abnormal changes in mortality, body weight, relative organ weights, hematology, and urinalysis in rats of both sexes (Tables 2–6 and Figure 2). Minor changes were found in some of the rats treated with BJIGT.

Because the liver plays a key role in the metabolism and excretion of chemical agents, it is the primary target organ for the toxic effects of agents. Liver discoloration may occur due to liver damage such as hepatitis [12], and this discoloration was observed in one of the male rats that received BJIGT at 1000 mg/kg/day. However, BJIGT did not induce significant changes in serum biochemical parameters of liver function such as albumin, hepatocyte integrity (AST, ALT, and GGT), or biliary system disorder (ALP and bilirubin). Furthermore, this finding occurred with a lower incidence and did not show a dose-dependent correlation. Thus, liver discoloration without correlation of serum analysis may result from the individual difference and is not a sign of toxicity.

In female rats, increased levels of Cl or Na were observed following administration of BJIGT at a dose of 500 mg/kg/day or 2000 mg/kg/day, respectively. The normal reference ranges for Cl and Na in female Crl:CD SD rats are as follows: 103.00–107.00 mmol/L for Cl and 141.00–149.00 mmol/L for Na [13]. Changes in Cl and Na levels did not exceed the normal range expected for these substances. The phosphorus level test may be used in the diagnosis of various bone and renal diseases, along with other tests including calcium, parathyroid hormone (PTH), and/or vitamin D. Although administration of BJIGT at a dose of 500 mg/kg/day increased the serum inorganic phosphorus (IP) level, it did not affect serum Ca level or the weights of the thymus, thyroid, and parathyroid glands. Change of IP level was detected only in females and was within the mean control range [14]. Thus, these changes in serum analysis were not considered to be BJIGT-mediated abnormalities.

## 5. Conclusions

Our findings demonstrate that oral administration of BJIGT did not exert any toxic effects in both sexes of Crl:CD SD rats over a 4-week period. The NOAELs of BJIGT are determined up to 2000 mg/kg/day regardless of sex. Further investigations, including a chronic toxicity study and a genotoxicity study, are needed to establish the safety and toxicity profile of BJIGT.

## Figures and Tables

**Table 1:** Amounts of eight marker components in the BJIGT samples at 0, 1, and 4 weeks by HPLC (*n* = 3).

Compound	0 weeks			1 week			4 weeks		
	Mean (mg/g)	SD	RSD (%)	Mean (mg/g)	SD	RSD (%)	Mean (mg/g)	SD	RSD (%)
Liquiritin	1.90	0.02	1.28	1.90	0.03	1.41	1.90	0.03	1.52
Nodakenin	1.28	0.01	0.72	1.24	0.02	1.55	1.23	0.01	1.17
Hesperidin	1.73	0.03	1.53	1.63	0.02	1.20	1.65	0.02	1.28
Glycyrrhizin	4.69	0.04	0.92	4.63	0.07	1.59	4.52	0.04	0.93

**Table 2:** Mortality and clinical signs in rats treated with BJIGT for 4 weeks.

Group	Dosing phase					Scratch wound, slight^b^
	1 day	≤1 week	≤2 weeks	≤4 weeks	Final mortality^a^	
Male						
0 mg/kg/day	0	0	0	0	0/5	2/5
500 mg/kg/day	0	0	0	0	0/5	0/5
1000 mg/kg/day	0	0	0	0	0/5	0/5
2000 mg/kg/day	0	0	0	0	0/5	0/5
Female						
0 mg/kg/day	0	0	0	0	0/5	0/5
500 mg/kg/day	0	0	0	0	0/5	0/5
1000 mg/kg/day	0	0	0	0	0/5	0/5
2000 mg/kg/day	0	0	0	0	0/5	0/5

^a^Number of animals with dead animals/total animal number. ^b^Number of animals with sign animals/total animal number.

**Table 3:** Hematological parameters of rats treated with BJIGT for 4 weeks.

Dose (mg/kg/day)	0	500	1000	2000
*Male*				
WBC (10^3^/*μ*L)	9.97 ± 0.86	11.33 ± 3.88	10.12 ± 0.88	11.63 ± 3.08
RBC (10^6^/*μ*L)	8.21 ± 0.43	8.36 ± 0.33	8.03 ± 0.25	8.10 ± 0.36
HGB (g/dL)	15.6 ± 0.85	16.0 ± 0.58	15.6 ± 0.70	15.7 ± 0.38
HCT (%)	48.8 ± 2.12	50.4 ± 1.95	4.90 ± 2.44	48.9 ± 1.19
MCV (fL)	59.5 ± 0.61	60.4 ± 1.59	61.1 ± 2.27	60.5 ± 1.91
MCH (pg)	19.0 ± 0.35	19.1 ± 0.22	19.5 ± 0.67	19.4 ± 0.67
MCHC (g/dL)	31.9 ± 0.55	31.7 ± 0.46	31.9 ± 0.60	32.1 ± 0.22
RET (%)	2.52 ± 0.63	2.40 ± 0.33	2.77 ± 0.51	2.77 ± 0.19
PLT (10^3^/*μ*L)	1010.4 ± 133.68	985.6 ± 45.00	109.4 ± 110.75	935.8 ± 134.33
PT (sec)	13.2 ± 0.56	13.0 ± 0.68	13.0 ± 0.41	12.6 ± 0.43
APTT (sec)	17.9 ± 0.76	18.5 ± 1.44	17.3 ± 0.72	17.7 ± 0.58
Neutrophils (%)	11.4 ± 3.97	10.4 ± 2.60	10.3 ± 1.69	12.3 ± 2.42
Lymphocytes (%)	84.7 ± 4.280	84.9 ± 3.28	84.9 ± 2.20	83.3 ± 2.84
Eosinophils (%)	0.70 ± 0.330	0.70 ± 0.210	0.70 ± 0.230	0.70 ± 0.360
Monocytes (%)	2.30 ± 0.530	2.40 ± 0.780	2.40 ± 0.520	2.30 ± 0.620
Basophils (%)	0.30 ± 0.050	0.30 ± 0.050	0.30 ± 0.110	0.30 ± 0.040
LUC (%)	0.70 ± 0.190	1.40 ± 0.610	1.40 ± 0.510	1.00 ± 0.190
*Female*				
WBC (10^3^/*μ*L)	7.62 ± 2.30	6.58 ± 1.42	8.09 ± 2.32	7.59 ± 2.55
RBC (10^6^/*μ*L)	8.18 ± 0.36	8.45 ± 0.57	8.18 ± 0.48	8.25 ± 0.49
HGB (g/dL)	15.8 ± 0.73	16.4 ± 0.79	15.9 ± 0.64	15.6 ± 0.68
HCT (%)	48.3 ± 2.08	50.0 ± 2.43	48.40 ± 1.82	47.6 ± 1.95
MCV (fL)	59.1 ± 0.65	59.2 ± 1.56	59.2 ± 2.80	57.8 ± 1.32
MCH (pg)	19.3 ± 0.22	19.5 ± 0.56	19.5 ± 0.91	18.9 ± 0.42
MCHC (g/dL)	32.7 ± 0.39	32.9 ± 0.11	32.9 ± 0.19	32.7 ± 0.19
RET (%)	2.16 ± 0.42	2.40 ± 0.32	2.37 ± 0.48	2.20 ± 0.40
PLT (10^3^/*μ*L)	999.8 ± 140.50	1041.6 ± 126.09	974.6 ± 73.23	999.6 ± 137.43
PT (sec)	12.7 ± 0.51	12.9 ± 0.29	12.8 ± 0.41	12.6 ± 0.27
APTT (sec)	14.8 ± 1.33	14.3 ± 1.86	15.2 ± 2.05	15.4 ± 1.04
Neutrophils (%)	9.1 ± 2.91	11.0 ± 3.02	8.5 ± 2.62	9.8 ± 2.97
Lymphocytes (%)	86.8 ± 4.03	84.8 ± 2.71	88.0 ± 2.39	86.3 ± 2.68
Eosinophils (%)	0.80 ± 0.300	1.00 ± 0.510	0.70 ± 0.340	1.00 ± 0.340
Monocytes (%)	2.10 ± 0.910	1.80 ± 0.740	1.60 ± 0.650	1.70 ± 0.810
Basophils (%)	0.30 ± 0.190	0.30 ± 0.090	0.20 ± 0.100	0.20 ± 0.090
LUC (%)	1.00 ± 0.300	1.10 ± 0.260	1.00 ± 0.360	1.00 ± 0.190

Results are presented as mean ± SD. WBC, white blood cell; RBC, total red blood cell; HGB, hemoglobin; HCT, hematocrit; MCV, mean corpuscular volume; MCH, mean corpuscular hemoglobin; MCHC, mean corpuscular hemoglobin concentration; RET; reticulocyte count, PLT; platelet count, PT; prothrombin time; APTT; activated partial thromboplastin time; LUC, large unstained cells.

**Table 4:** Serum biochemical parameters of male rats treated with BJIGT for 4 weeks.

Dose (mg/kg/day)	0	500	1000	2000	0	500	1000	2000
	*Male*				*Female*			
Glucose (mg/dL)	129.8 ± 43.02	122.3 ± 24.93	128.4 ± 22.43	105.8 ± 17.39	94.6 ± 24.89	83.6 ± 27.68	88.7 ± 29.32	123.5 ± 26.52
BUN (mg/dL)	14.8 ± 1.60	15.9 ± 2.23	15.2 ± 1.72	15.20 ± 1.80	19.00 ± 2.91	19.10 ± 1.60	19.30 ± 2.11	16.50 ± 1.10
Creatinine (mg/dL)	0.47 ± 0.04	0.53 ± 0.07	0.46 ± 0.04	0.51 ± 0.05	0.51 ± 0.03	0.51 ± 0.03	0.52 ± 0.05	0.48 ± 0.04
Creatine kinase (IU/L)	658.2 ± 112.01	689.6 ± 251.21	655.4 ± 135.68	791.6 ± 282.74	792 ± 114.20	788.6 ± 198.62	577.2 ± 108.40	698.4 ± 221.97
Total protein (g/dL)	6.15 ± 0.49	6.40 ± 0.25	6.27 ± 0.30	6.05 ± 0.17	6.80 ± 0.28	6.54 ± 0.27	6.78 ± 0.11	7.12 ± 0.47
Albumin (g/dL)	4.13 ± 0.26	4.23 ± 0.14	4.09 ± 0.17	4.01 ± 0.14	4.57 ± 0.16	4.37 ± 0.17	4.52 ± 0.15	4.74 ± 0.26
Albumin/globulin ratio	2.06 ± 0.19	1.95 ± 0.08	1.88 ± 0.09	1.97 ± 0.07	2.06 ± 0.08	2.02 ± 0.04	2.00 ± 0.14	2.01 ± 0.16
AST (IU/L)	142.7 ± 14.04	137.4 ± 21.15	133.9 ± 7.35	145.0 ± 19.91	150.6 ± 14.71	142.9 ± 16.92	127.9 ± 9.96	137.0 ± 18.96
ALT (IU/L)	33.2 ± 7.69	33.3 ± 4.12	33.3 ± 3.50	29.9 ± 3.98	25.8 ± 4.47	25.0 ± 1.92	25.7 ± 2.93	23.9 ± 4.66
GGT (IU/L)	0.10 ± 0.11	0.21 ± 0.18	0.42 ± 0.22	0.35 ± 0.19	1.03 ± 0.31	0.66 ± 0.21	0.92 ± 0.23	0.82 ± 0.30
Total bilirubin (mg/dL)	0.117 ± 0.016	0.109 ± 0.013	0.117 ± 0.018	0.121 ± 0.015	0.144 ± 0.006	0.130 ± 0.019	0.147 ± 0.014	0.130 ± 0.019
Total cholesterol (mg/dL)	52.4 ± 9.18	50.8 ± 8.07	60.6 ± 10.97	59.6 ± 15.31	59.8 ± 28.10	51.4 ± 14.84	55.8 ± 5.40	59.8 ± 17.08
Triglycerides (mg/dL)	17.7 ± 4.44	1.30 ± 6.49	19.7 ± 8.62	19.2 ± 4.03	8.60 ± 1.32	8.80 ± 1.37	8.30 ± 2.15	10.40 ± 1.57
Phospholipid (mg/dL)	85.8 ± 10.57	83.0 ± 5.83	97.6 ± 10.38	91.4 ± 18.12	112.4 ± 40.02	99.2 ± 20.68	107.0 ± 7.81	116.0 ± 22.66
ALP (IU/L)	722.4 ± 117.44	618.1 ± 82.69	749.9 ± 186.08	687.1 ± 110.64	431.7 ± 71.09	401.8 ± 52.32	364.5 ± 88.76	409.8 ± 116.39
Ca (mg/dL)	10.81 ± 0.45	10.71 ± 0.25	10.82 ± 0.44	10.55 ± 0.36	10.72 ± 0.31	10.74 ± 0.43	10.85 ± 0.25	11.08 ± 0.38
IP (mg/dL)	10.79 ± 1.42	10.56 ± 0.31	10.67 ± 0.58	9.93 ± 0.29	9.14 ± 0.41	9.76^*∗*^ ± 0.37	9.16 ± 0.30	9.46 ± 0.35
K (mmol/L)	7.93 ± 1.66	7.22 ± 0.94	6.87 ± 0.73	6.94 ± 0.98	7.03 ± 0.63	7.86 ± 0.20	6.95 ± 0.94	6.34 ± 0.83
Na (mmol/L)	141.6 ± 2.51	142.8 ± 1.30	14.3 ± 1.22	143.2 ± 1.48	141.4 ± 1.52	141.8 ± 1.30	142.6 ± 1.14	143.6^*∗*^ ± 0.55
Cl (mmol/L)	102.0 ± 1.73	103.2 ± 1.30	102.2 ± 1.64	103.2 ± 1.30	102.8 ± 1.48	105.6^*∗*^ ± 1.67	105.2 ± 1.79	103.2 ± 1.30

Results are presented as mean ± SD. *∗* indicates significance at the *p* < 0.05 level. BUN, blood urea nitrogen; CK, creatine kinase; AG, albumin/globulin ratio; AST, aspartate aminotransferase; ALT, alanine aminotransferase; GGT, gamma glutamyl transpeptidase; TB, total bilirubin; TCHO, total cholesterol; TG, triglyceride; PL, phospholipid; ALP, alkaline phosphatase; IP, inorganic phosphorus.

**Table 5:** Urinalysis parameters of female rats treated with BJIGT for 4 weeks.

Dose (mg/kg/day)	0	500	1000	2000
*Male*				
Volume (mL)	17.8 ± 6.42	14.8 ± 3.11	22.8 ± 7.89	15.4 ± 5.50
Specific gravity	1.012 ± 0.005	1.013 ± 0.003	1.012 ± 0.003	1.015 ± 0.004
pH	6.8 ± 0.27	7.0 ± 0.00	6.9 ± 0.22	6.6 ± 0.22
*Female*				
Volume (mL)	15.2 ± 5.07	15.6 ± 7.96	15.0 ± 6.71	10.6 ± 3.58
Specific gravity	1.015 ± 0.004	1.014 ± 0.007	1.015 ± 0.005	1.016 ± 0.002
pH	6.5 ± 0.50	6.7 ± 0.27	6.5 ± 0.50	6.5 ± 0.00

Results are presented as mean ± SD.

**Table 6:** Relative organ weight (%) of rats treated with BJIGT for 4 weeks.

Dose (mg/kg/day)	0	500	1000	2000
*Male*				
Body weight	353.7 ± 37.00	343.4 ± 23.84	366.7 ± 36.86	345.5 ± 20.41
Brain	0.554 ± 0.0314	0.602 ± 0.0480	0.551 ± 0.0467	0.567 ± 0.0274
Heart	0.352 ± 0.0308	0.337 ± 0.0217	0.347 ± 0.0345	0.335 ± 0.0169
Lung	0.403 ± 0.0137	0.369 ± 0.0200	0.382 ± 0.0173	0.402 ± 0.0310
Kidneys	0.834 ± 0.0362	0.857 ± 0.0394	0.830 ± 0.0391	0.822 ± 0.0363
Liver	3.177 ± 0.1853	3.247 ± 0.1692	3.252 ± 0.1247	3.258 ± 0.2019
Spleen	0.213 ± 0.0362	0.183 ± 0.0205	0.197 ± 0.0293	0.220 ± 0.0318
Testes	0.881 ± 0.1197	0.887 ± 0.0633	0.835 ± 0.0436	0.827 ± 0.0852
Prostate	0.127 ± 0.0168	0.128 ± 0.0083	0.120 ± 0.0253	0.135 ± 0.0069
Epididymis	0.260 ± 0.0196	0.259 ± 0.0331	0.247 ± 0.0282	0.279 ± 0.0348
Thymus	0.134 ± 0.0136	0.137 ± 0.0279	0.142 ± 0.0179	0.145 ± 0.0157
THPA	0.007 ± 0.0013	0.007 ± 0.0012	0.006 ± 0.0014	0.007 ± 0.0014
PITG	0.003 ± 0.0002	0.004 ± 0.0006	0.004 ± 0.0011	0.004 ± 0.0011
ADRG	0.016 ± 0.0013	0.016 ± 0.0023	0.015 ± 0.0010	0.016 ± 0.0013
SVCG	0.352 ± 0.0601	0.350 ± 0.0491	0.346 ± 0.1059	0.366 ± 0.0677
SALG	0.184 ± 0.0181	0.208 ± 0.0090	0.177 ± 0.0226	0.184 ± 0.0219
*Female*				
Body weight	215.0 ± 10.75	218.9 ± 21.44	215.9 ± 13.58	221.0 ± 18.29
Brain	0.841 ± 0.0534	0.867 ± 0.0746	0.863 ± 0.0425	0.863 ± 0.0482
Heart	0.376 ± 0.0215	0.353 ± 0.0253	0.381 ± 0.0403	0.353 ± 0.0179
Lung	0.523 ± 0.0312	0.523 ± 0.0312	0.514 ± 0.0585	0.523 ± 0.0258
Kidneys	0.919 ± 0.0655	0.845 ± 0.0353	0.868 ± 0.0820	0.929 ± 0.0595
Liver	3.359 ± 0.2068	3.266 ± 0.1304	3.931 ± 0.0920	3.439 ± 0.1393
Spleen	0.209 ± 0.0111	0.213 ± 0.0308	0.214 ± 0.0245	0.200 ± 0.0198
Ovaries	0.041 ± 0.0057	0.040 ± 0.0061	0.043 ± 0.0064	0.042 ± 0.0072
UTEC	0.206 ± 0.0432	0.206 ± 0.0432	0.239 ± 0.0529	0.235 ± 0.0500
Thymus	0.182 ± 0.0294	0.210 ± 0.0321	0.192 ± 0.0331	0.203 ± 0.0247
THPA	0.009 ± 0.0018	0.009 ± 0.0018	0.007 ± 0.0014	0.008 ± 0.0013
PITG	0.007 ± 0.0018	0.007 ± 0.0012	0.007 ± 0.0010	0.007 ± 0.0005
ADRG	0.034 ± 0.0053	0.033 ± 0.0046	0.036 ± 0.0022	0.035 ± 0.0049
SALG	0.190 ± 0.0070	0.190 ± 0.0173	0.215 ± 0.0190	0.215 ± 0.0225

Results are presented as mean ± SD. THPA, thyroid and parathyroid glands; PITG, pituitary gland; ADRG, adrenal glands; SVCG, seminal vesicles with coagulating gland; SALG, salivary glands; UTEC, uterus/cervix.
